# Notes and News

**Published:** 1892-07-09

**Authors:** 


### Notes and News.

COLLECTED AND COMMUNICATED.

An interesting exhibition and sale of work done by the blind and decrepit inmates of workhouses was held at Lancaster Gate last week, through the kindness of Lady Meath. The exhibition was both interesting and praiseworthy, and, if it is the means of giving a little zest to lives necessarily too frequently sad and monotonous, it will have accomplished a kindly and useful work.

The board-room at St. Mary's Hospital was the scene of an interesting little ceremony last week. This was the presentation of the St. John Ambulance certificates for first aid to the sick to cab shelter attendants of the Street Ambulance Corps, by Mrs. Bischoffsheim. No one more welcome could have been chosen for the office than the lady in question, for as the wife of Mr. Bischoffsheim, the Treasurer and most generous supporter of the movement, she appealed to the sympathy of all present. The Street Ambulance is a branch of the Hospitals Association, and has been established for about three years. The work has been unostentatiously and effectively accomplished, principally through the energy and good organisation of Mr. Ryan. So soon as the scheme was proved to be fulfilling an urgent need, and to being also efficiently conducted, Mr. Bischoffsheim undertook the whole of the financial responsibility. Few citizens have rendered better service to the metropolis than this. There are now very numerous stations, and during the past year over 1,000 persons suffering from accidents have been conveyed to hospitals or elsewhere. A most effective corps has been formed by the attendants of the cabmen's shelters, who responded to an invitation to attend classes for instruction in first aid to the sick, with the excellent result that out of the whole number only one member failed to pass the examination. In presenting the certificates Mrs. Bischoffsheim made a graceful little speech, which was enthusiastically received. She was supported by Mr. Bischoffsheim, Dr. Bristowe, Mr. Ryan, Mr. Burdett, and others.

The second International Congress of Physiology will be held at Liége on August, 28th, 29th, 30th, and 31st next. The director of the Physiological Institute at Liége is ready to supply all information respecting arrangements. The Anthropological Society of Germany will hold its annual congress at Ulm, on July 31st.

The microscope of science is focused upon street sweepings with a vengeance. A Neapolitan doctor has subjected the sweepings of Naples to close scrutiny, and publishes as a result that every gramme of this heterongeneous material contains over 910,000 living bacteria. The dirt of Naples is proverbial, but we sincerely trust that in this case it is not quite so lively as it is reported.

Charming summer numbers are issued by the *Leisure Hour* Office of the Girl's and Boy's Own Papers. The frontispiece of the *Boy's Own Paper* is especially attractive. In it the "Lost Chord" is represented by a fine figure, half warlike, half mythological, drawn in a fine brown on a blue ground. Within the cover, "Molly" is likely to prove a favourite story. Be it understood that "Molly" is not a girl.

It is impossible to overlook the fact any longer that scarlet fever is unpleasantly prevalent in the metropolis and immediately surrounding districts. We hear that the Metropolitan Asylums Board fever hospitals are full, and that huts are rapidly being built in connection with them. Cases are said to be coming in at the rate of fifty a day, and the nursing staffs at the hospitals are sadly overtaxed.

We are very glad to learn that Lady Meath has succeeded in her scheme for a home for epileptics at Godalming. The want of such an institution has been pointed out to us from numerous sources lately, and we feel sure that the foundress will have every reason to be encouraged by the fact that she is fulfilling a pressing want. The home to be opened in August will receive female inmates only, and we notice that members of the G.F.S., in whom Lady Meath takes so warm an interest, are to be received on specially favourable terms. The success of the Brabazon Convalescent Home augurs well for this latest undertaking of Lady Meath's.

The foundation of the Toxteth Poor Law Infirmary, Liverpool, is laid at last, after much difficulty and vexation of spirit. Apparently the promoters do not expect to "be happy ever after," if we may judge from a remark made by the senior member of the Board of Guardians, who in laying the foundation stone said that as the enlargement of the hospital had been in some measure forced upon the guardians, he trusted the ratepayers would in the true spirit of English generosity allow them to prosecute the scheme without adverse criticism. The new buildings are to cost £28,000, and the infirmary is to contain 420 beds. The premises are to consist of blocks, pavilions, and nurses' home. The pavilions are to number seven, of two storeys each.

